# Effect of Meperidine on Equine Blood Histamine, Tryptase, and Immunoglobulin-E Concentrations

**DOI:** 10.3389/fvets.2020.584922

**Published:** 2020-12-23

**Authors:** H. Nicole Trenholme, Daniel M. Sakai, Londa J. Berghaus, Amanda L. Hanafi, Heather K. Knych, Clare A. Ryan, Brittany McHale, Frane Banovic, Jane E. Quandt, Michele Barletta, Rachel A. Reed

**Affiliations:** ^1^Department of Large Animal Medicine, College of Veterinary Medicine, University of Georgia, Athens, GA, United States; ^2^Department of Small Animal Medicine and Surgery, College of Veterinary Medicine, University of Georgia, Athens, GA, United States; ^3^Peterson and Smith Equine Hospital, Ocala, FL, United States; ^4^K.L. Maddy Equine Analytical Pharmacology Laboratory, School of Veterinary Medicine, University of California, Davis, Davis, CA, United States; ^5^Department of Molecular Biosciences, School of Veterinary Medicine, University of California, Davis, Davis, CA, United States; ^6^Infectious Disease Laboratory, College of Veterinary Medicine, Department of Small Animal Medicine and Surgery, University of Georgia, Athens, GA, United States

**Keywords:** anaphylactoid, anaphylaxis, histamine, horse, meperidine, opioid, tryptase, immunoglobulin E

## Abstract

**Objectives:** To evaluate changes in immunological parameters following subcutaneous (SC) and intramuscular (IM) administration of meperidine in horses through quantitative analysis of plasma tryptase, histamine, and IgE levels.

**Methods:** Six adult horses were enrolled in a prospective randomized crossover design. Horses were administered one treatment per day, with a seven day washout period: (a) meperidine 1 mg/kg IM, saline 6 mL SC; (b) saline 6 mL IM, meperidine 1 mg/kg SC; (c) saline 6 mL SC, saline 6 mL IM. Blood samples were obtained for plasmatic histamine (baseline, 5, 10, 15, 30, and 60 min) via LC-MS/MS and plasmatic tryptase (baseline, 15, 30, 60, 120, and 240 min) quantification with enzyme-linked immunoabsorbent assays. Serum immunoglobulin E (IgE) concentrations prior to any meperidine treatment and 7–14 days following the first meperidine treatment were evaluated with enzyme-linked immunoabsorbent assays. Histamine and tryptase concentrations were evaluated with a mixed-effect analysis of variance. The levels of IgE at baseline (before the administration of the first dose of meperidine) were compared with the IgE values at 60 min following the second meperidine administration with the Paired *t* test. Biopsies of localized injection site reactions from subcutaneous meperidine administration were collected from two horses.

**Results:** No statistically significant elevations from baseline in histamine (*p* = 0.595), tryptase (*p* = 0.836), or IgE (*p* = 0.844) were found in any of the horses in this study. There were no differences between treatment groups. Administration of SC meperidine caused a localized vasculitis and thrombosis with regional edema and hemorrhage.

**Conclusion:** No evidence of anaphylactoid or anaphylactic type reactions occurred following IM or SC meperidine administration.

## Introduction

Administration of opioid agonist agents is known to cause both type I hypersensitivity (anaphylactic) reactions and pseudoallergy (anaphylactoid) reactions (1). True anaphylactic reactions occur when immunoglobulin E (IgE), bound to cell surface receptors, encounters a specific antigen. Cross-linking of IgE receptors causes subsequent activation of mast cells, eosinophils, and basophils. Anaphylactic responses are considered rare after opiate administration (1–3). Pseudoallergy reactions, in contrast, require no prior sensitization and are more commonly observed in humans. They occur via non-IgE mediated mechanisms (4), where substances and antigens directly activate receptors on the mast cells with subsequent release of inflammatory mediators (5, 6). Specifically, mast cell degranulation causes the release of tryptase and histamine, initiating a systemic inflammatory response (7). These inflammatory responses are known to occur following the administration of morphine, meperidine, and codeine in humans (8, 9) and after hydromorphone and morphine in dogs (10, 11). The exact mechanism of opioid induced activation of mast cells is unknown at this time. Although mast cells express opioid receptors, recent studies revealed that Mas-related G protein-coupled receptor X2 might be the essential receptor for the transmission of opioid function in human skin mast cells (12–14). Clinically, anaphylactic and anaphylactoid responses in human patients are diagnosed using a combination of history, histamine levels, tryptase levels, evaluation of antigen-specific IgE, and skin-prick testing (15).

Meperidine is a pure mu opioid receptor agonist used in various species to provide analgesia (16–20). Reported adverse effects of intravenous (IV) administration of meperidine in horses include sweating, tachycardia, hypotension, and erythema (19, 21, 22). Such effects are presumed to be a result of mast cell degranulation, as meperidine is known to cause histamine release in dogs (23) and humans (24). However, this syndrome has not been confirmed in horses. Due to the incidence of severe adverse effects when meperidine is administered IV, many clinicians prefer to administer the drug either via intramuscular (IM) or subcutaneous (SC) routes. None of the above listed adverse effects observed after IV administration were noted in horses given IM meperidine at a dose of 1 mg/kg (19, 25). However, it is unknown whether administration of meperidine IM or SC in this species can cause subclinical release of inflammatory mediators characterized by elevations in histamine and tryptase plasma concentrations.

The aim of this study was to evaluate inflammatory mediator responses to meperidine administration in horses through quantitative analysis of plasma histamine and tryptase concentrations and serum IgE concentrations following a single 1 mg/kg IM or SC dose. The dose of 1 mg/kg was chosen based on the previous literature cited above in which 1 mg/kg was utilized and the clinical experience of the authors. The authors hypothesized that meperidine would increase both histamine and tryptase concentrations in horses due to an anaphylactoid response. Additionally, it was hypothesized that serum IgE concentrations would not change over the study period. To the authors' knowledge, this is the first study evaluating the effect of IM or SC meperidine on these immune parameters in any species.

## Materials and Methods

The study was approved by the University of Georgia's Institutional Care and Use Committee (AUP A2018 01-023).

### Animals

Six adult University owned research horses (four Quarter horses, two Thoroughbreds; three male, three female) deemed healthy based on physical exam, weighing 494 ± 33 kilograms, and aged 14.6 ± 7.4 years were enrolled. Using a masked, prospective, Latin square design, horses randomly received each of three treatments with a 7-day washout period.

### Care and Instrumentation

Prior to each study day, every subject was acclimatized to a climate controlled 12' × 12' stall for 12–24 h prior to treatment. Horses were fed 2.2 kg of senior feed and two flakes of Timothy hay twice daily. The morning of treatment, each horse was weighed, and a physical exam was performed to obtain baseline physiologic variables. The skin over the left jugular groove was clipped and aseptically prepared. A 14 gauge 8.25 cm over-the-needle catheter (Mila International, Inc., KY, USA) was placed in the left jugular vein for blood sampling.

### Treatment Administration

Treatments were as follows:

- Meperidine 1 mg/kg IM and 6 mL saline SC
- Saline 6 mL IM and meperidine 1 mg/kg SC
- Saline 6 mL IM and saline 6 mL SC.

Each meperidine (Meperidine HCl Injection 100 mg/mL, NJ, USA) treatment was prepared by an unmasked participant (RR) by diluting the drug with saline (0.9% Sodium Chloride injection 1,000 mL, Baxter Healthcare Corporation, IL, US) to a fixed volume of 6 mL in order to facilitate masking. Intramuscular and SC injections were administered by masked participants (HNT, AH) simultaneously over 60 s with the side of the IM injection (right or left) determined by a coin toss. The IM and SC injections were made within and superficial to, respectively, the trapezius muscle using a 22 gauge, 2.54 cm hypodermic needle (Covidien Monoject, Medtronic, MN, USA). Following treatment administration, injection sites were monitored hourly for evidence of adverse reactions by masked participants (HNT, AH).

### Plasma and Serum Sampling

Following withdrawal of a 10 mL waste sample, 12 mL of blood was obtained at baseline, 5, 10, 15, 30, 60, 120, and 240 min post treatment administration. Six mL of whole blood from baseline through 60 min were placed immediately into lithium heparin tubes for histamine and tryptase determination (Becton, Dickinson, and Company, NJ, USA) and 6 mL from all timepoints were placed into serum collection tubes for IgE determination (Becton, Dickinson, and Company, NJ, USA). Samples were then centrifuged at 1,300 *g* for 10 min. The plasma and serum were collected and stored at −80° Celsius in storage cryovials (VWR International, PA, USA). Following sample collection, the catheter was removed.

### Histamine Plasma Concentration Determination

Histamine plasma concentrations were determined at baseline, 5, 10, 15, 30, and 60 min after the administration of meperidine to capture the early rise and fall of histamine observed in an anaphylactoid type reaction.

Calibrators were prepared by dilution of the histamine working standard solutions (Toronto Research Chemicals, Toronto, ON) with Tris BSA as a surrogate plasma matrix, as previously described (26) to concentrations ranging from 2.5 to 100 ng/mL. Calibration curves and negative control samples were prepared fresh for each quantitative assay. In addition, quality control samples (Tris BSA fortified with analyte at three concentrations within the standard curve) were included with each sample set as an additional check of accuracy.

Prior to analysis, 0.5 mL of the plasma sample was diluted with 600 μL of acetonitrile (ACN) containing d4-histamine (Toronto Research Chemicals, Toronto, ON) internal standard at 0.01 ng/μL and 100 μL of Tris BSA, to precipitate proteins. The samples were vortexed for 2 min to mix, refrigerated for 20 min, vortexed for an additional minute and centrifuged (4,300 rpm/3,102 g) for 10 min at 4°C. The sample was subsequently injected into the liquid chromatography tandem mass spectrometry (LC-MS/MS) system.

The concentration of histamine was measured in plasma by positive mode LC-MS/MS. Quantitative analysis was performed on a TSQ Altis triple quadrupole mass spectrometer coupled with a Vanquish liquid chromatography system (Thermo Scientific, San Jose, CA). Chromatography employed an Atlantis HILIC 10 cm × 2.1 mm 3 mm column (Waters Corp, Milford, MA) and a linear gradient of ACN in water with a constant 0.2% formic acid at a flow rate of 0.5 ml/min. The initial ACN concentration was held at 95% for 0.6 min, ramped to 5% over 2.4 min and held at that concentration for 0.1 min, before re-equilibrating for 3.9 min at initial conditions.

Detection and quantification was conducted using selective reaction monitoring of initial precursor ion for histamine (mass to charge ratio, 112) and d4-histamine (mass to charge ratio, 116). The response for the product ions for histamine (mass to charge ratio, 95) and d4-histamine (mass to charge ratio, 72, 85, 99) were plotted and peaks at the proper retention time integrated using Quanbrowser software (Thermo Scientific, Waltham, MA, USA). Quanbrowser software was used to generate calibration curves and quantitate histamine in all samples by linear regression. A weighting factor of 1/X was used for all calibration curves.

The concentration-response relationship (relationship between calibrators and the LC-MS/MS instrument response) for histamine was linear and provided a correlation coefficients of 0.99 or higher. The precision and accuracy of the assay were determined by assaying quality control samples in replicates (*n* = 6) for histamine. Accuracy was reported as percent nominal concentration and precision was reported as percent relative standard deviation. For histamine, accuracy was 115% for 3 ng/mL, 95% for 10 ng/mL and 104% for 50 ng/mL. Precision was 1% for 3 ng/mL, 5% for 10 ng/mL and 3% for 50 ng/mL. The technique was optimized to provide a limit of quantitation of 1 ng/mL and a limit of detection of approximately 0.5 ng/mL for histamine.

### Tryptase Plasma Concentration Determination

Plasma tryptase levels were determined at time points baseline, 15, 30, 60, 120, and 240 min in order to capture the first several hours following administration of meperidine as this is when tryptase would most likely be elevated.

Plasma concentration was determined using a quantitative sandwich enzyme-linked immunoabsorbent assays (ELISA) [Equine Tryptase Beta 2 (TPSB2) ELISA Kit, My Bio Source, CA, USA] performed according to manufacturer recommendations. All contents of the kit were allowed to reach room temperature. Standards contained in the kit represented a concentration gradient of 2-fold dilutions (ng/mL): 8, 4, 2, 1,.5, and 0.25. Duplicates of each standard and sample were added to pre-coated plates included in the kit, and duplicate blank wells were left empty according to manufacturer's guidelines. Then 100 μL of HRP-Conjugate reagent was added to standard and sample wells and the plate was incubated for 60 min at 37°C. The plate was washed 4 times with 1x wash solution (made from 20X Wash solution stock diluted in distilled water). Fifty μL of Chromagen Solution A followed by 50 μL of Chromogen Solution B was added to every well (including blank wells), and the plate was incubated in the dark for 15 min at 37° Celsius. Afterward, 50 μL of Stop Solution was added to each well and mixed with the contents. The plates were then placed on a microplate reader and optical density was measured at 450 nm for quantification (27).

The detection range was 0.25–8 ng/mL with a sensitivity of 0.1 ng/mL. Intra-essay and inter-assay coefficients of variation were <15%.

### Equine IgE Serum Concentration Determination

Serum IgE concentrations were determined at baseline, 5, 10, 15, 30, and 60 min following administration of meperidine aimed at capturing the timepoints in which a rapid anaphylactic reaction would occur.

A quantitative sandwich ELISA (Nori Equine IgE ELISA, Genorise Scientific, Inc, PA, USA) was performed according to manufacturer recommendations, with a detection range of 0.78–50 ng/mL (28). This test has been utilized elsewhere on serum samples (29). Assay buffer in the amount of 500 μL was added to one vial of Equine IgE standard, to make a 50 ng/mL concentration. Two-fold serial dilutions were made to generate a seven-point standard curve (50 to 0.78 ng/mL). Serum samples were diluted 1:4 according to manufacturer's guidelines. Then 100 μL of either the diluted sample or the Equine IgE Standard were aliquoted into duplicate wells of the pre-coated 96 well plate, and incubated at room temperature for 60 min. Wells were aspirated and washed 2 times with Assay Buffer solution. Then 100 μL of the Detection Antibody Solution was added to each well, and plates were incubated in the dark for 60 min at room temperature followed by two wash cycles. HRP-Conjugate in the amount of 100 μL was added to each well, and the plate was incubated for 20 min in the dark at room temperature, followed by a final two washes. Then 100 μL of substrate solution was added to each well, with a 20 min incubation period. The reaction was stopped by the addition of 50 μL of Stop Solution to each well. The plates were read at 450 nm (with wavelength correction set at 540 nm) for quantification (28).

The detection range was 0.5–50 ng/mL with a sensitivity of 0.1 ng/mL and an intra-assay coefficient of variation of 6% and inter-assay coefficient of variation of 9%.

### Biopsies

To evaluate a cutaneous reaction to meperidine injections, one 6-mm punch biopsy (Miltex, Integra Life Sciences Corporation, NJ, USA) was collected from two horses that received the subcutaneous meperidine treatment as their final treatment in the randomization. Only those two horses receiving the subcutaneous treatment as their last treatment in the randomization were biopsied for several reasons. First, the local reaction to the subcutaneous administration was not initially recognized as something that would happen in every horse as it had never been reported previously in the literature. Therefore, it was originally thought to be a reaction unique to the first horses receiving this treatment. Second, an amendment to the animal use protocol was required in order to obtain the biopsies, and this takes time. Lastly, it was unclear what effect, if any, that obtaining the biopsy would have on future treatments for a given horse in relation to the need for sedation and the presence of a biopsy sight in the area of injection for additional treatments. For those two horses which were biopsied, twenty-four hours following treatment administration, these horses were sedated with xylazine (100 mg; AnaSed, Akorn, Inc, IA, USA) for the biopsy procedure. The tissue sample was placed in 10% neutral buffered formalin for paraffin embedding and routine histopathology. Five-micrometer sections were cut from paraffin blocks and stained with haematoxylin and eosin for examination of inflammatory cells and any abnormalities. A single cutaneous suture (2–0 Ethilon, Ethicon US LLC, GA, USA) was placed to close the wound edges. The biopsy site was visually evaluated daily for the following 5 days, and sutures removed following healing, 10–14 days later.

### Statistical Analysis

Statistical analysis were performed using GraphPad (GraphPad Software, San Diego, CA, USA). Normal or lognormal distribution of baseline values were analyzed with the Shapiro-Wilk test. A mixed-effect ANOVA, with time and treatment as fixed effects and subject as random effect, was used to analyze histamine and tryptase concentrations. Values below limits of quantification were excluded from the analysis. Dunnett corrections were performed for multiple comparisons when necessary. The levels of IgE at baseline (before the administration of the first dose of meperidine) were compared with the IgE values at 60 min following the second meperidine administration with the paired *t* test. Alpha was set at 0.05.

## Results

**Figure 1:**
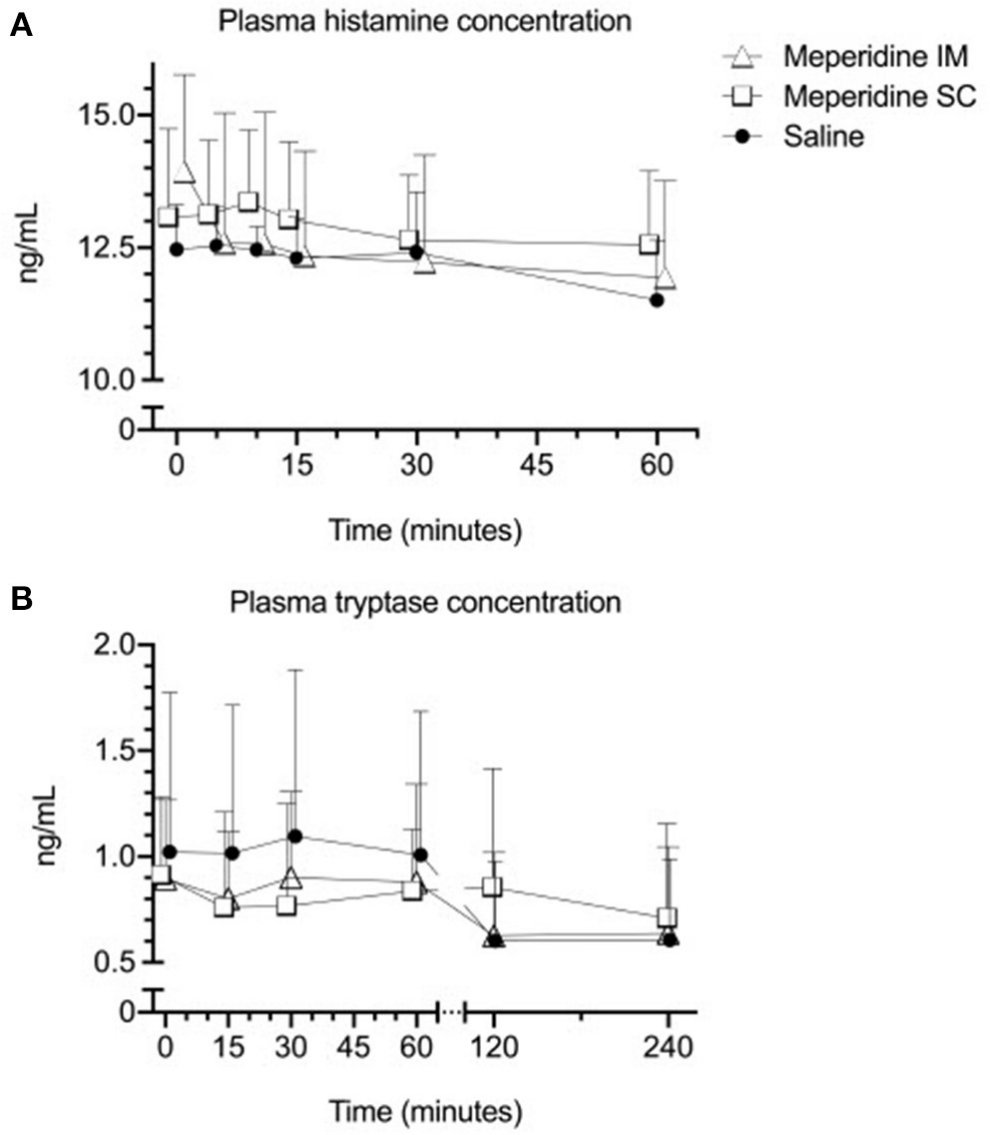
Mean ± SD plasma histamine **(A)** and tryptase **(B)** in horses (*n* = 6) following administration of 1 mg/kg meperidine intramuscularly (IM), 1 mg/kg meperidine subcutaneously (SC), or saline at baseline (time 0) and various time points following administration.

Plasma histamine concentrations ranged from 11.23 to 15.69 ng/mL (mean ± SD: 13.06 ± 1.48 ng/mL) at baseline. There was no significant effect of time (*p* > 0.061, for all after multiple comparisons) on plasma histamine concentration following treatment. There was no significant effect of treatment (*p* = 0.595) on plasma histamine concentration (Figure 1A). There was no significant effect of the interaction between time and treatment (*p* = 0.650) on plasma histamine concentration.

The plasma tryptase concentration ranged from < 0.250 to 1.943 ng/mL (0.948 ± 0.516 ng/mL) at baseline. There was no effect of time (*p* = 0.330) or treatment (*p* = 0.710) on plasma tryptase concentration (Figure 1B). There was no significant effect of the interaction between time and treatment (*p* = 0.597) on plasma tryptase concentration.

The serum IgE concentrations ranged from <0.50 to 35.13 ng/mL (13.70 ± 15.16 ng/mL) at baseline prior to administration of the first meperidine treatment. Sixty minutes following the second meperidine treatment, serum IgE concentrations ranged from <0.50 to 39.12 ng/mL (14.10 ± 16.42 ng/mL). There was no significant difference between baseline and IgE levels 60 min following the second meperidine treatment (*p* = 0.742). A period of at least 7–14 days elapsed between the first and second meperidine treatments. There was no significant effect of the interaction between time and treatment (*p* = 0.434) on plasma histamine concentration.

**Figure 2:**
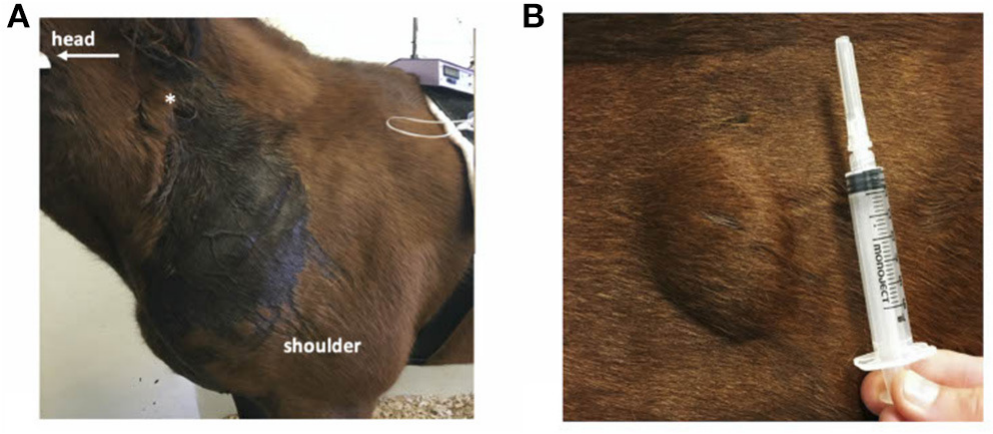
Cutaneous reactions of a horse administered subcutaneous meperidine (1 mg/kg). **(A)** Extensive localized sweating near site of injection (*). **(B)** Firm swelling at the site of injection.

All horses had cutaneous eruptions after subcutaneous administration of meperidine. These lesions were visible within 5–10 min as a firm raised wheal with regional sweating that radiated from the SC meperidine injection site (Figure 2). These eruptions were not anticipated at the onset of the study, and measurements of the dimensions of the eruptions were initiated when the pattern was recognized. The dimensions of the lesions are described in Table 1. There was no cutaneous reaction observed with meperidine IM or saline.

**Table 1:** Dimensions of cutaneous eruptions observed following subcutaneous administration of 1 mg/kg meperidine in six horses.

**Horse**	**Initial Swelling Size (mm)**	**Largest Swelling size (mm)**
1	Not measured	Not measured
2	Not measured	Not measured
3	Not measured	80 × 69 × 10
4	72 × 38 × 9	196 × 67.5 × 4.5
5	55 × 41 × 10	104 × 60 × 10
6	36 × 36 × 8	80 × 137 × 10

**Figure 3:**
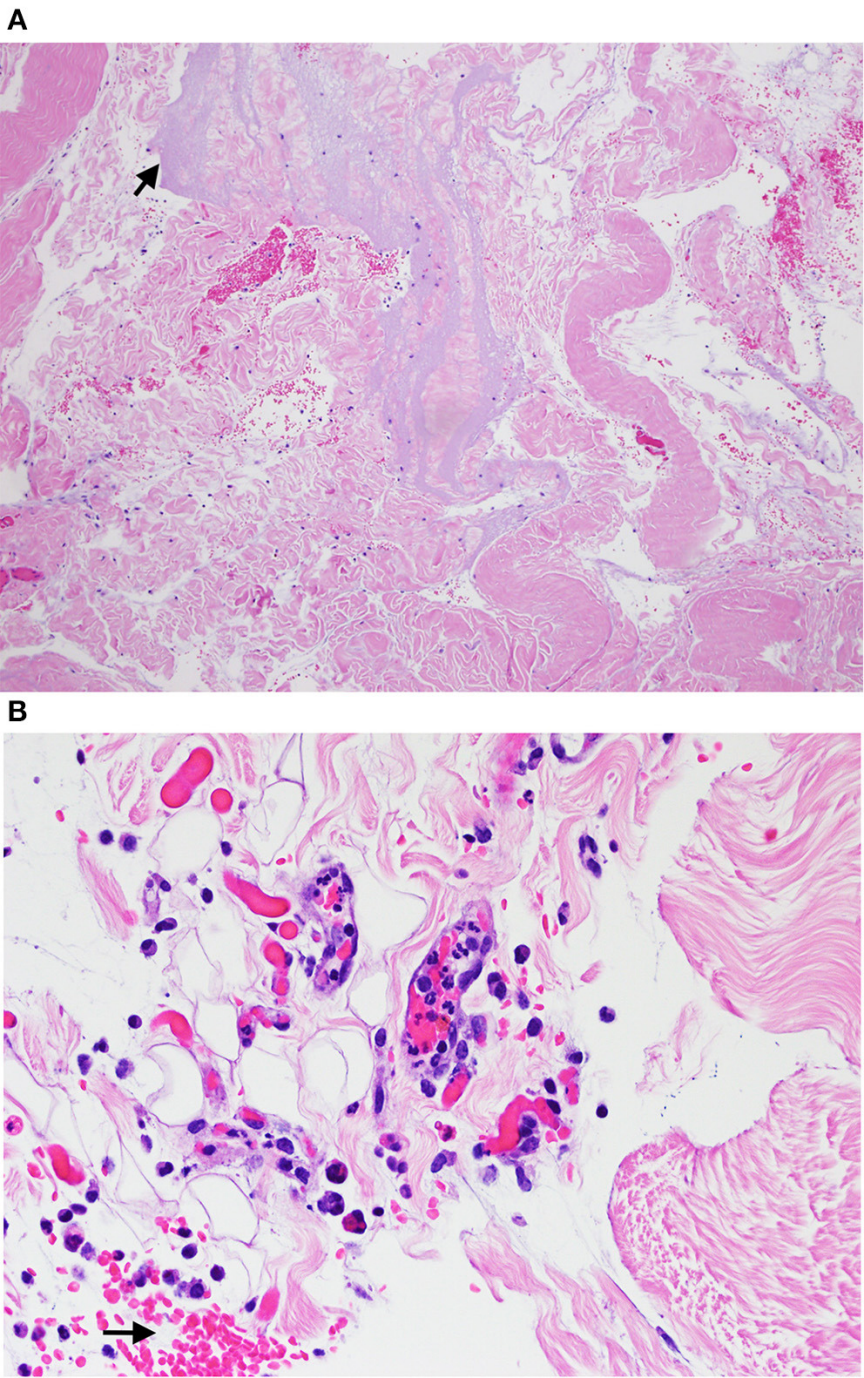
Hematoxylin and eosin stain section from biopsy samples obtained from cutaneous lesions in horses given subcutaneous meperidine (1 mg/kg) 24 h earlier visualized with light microscopy at 10x **(A)** and 40x **(B)**. **(A)** Arrow is pointing to amphilic material (suspect injection material) that expanded in the dermis. **(B)** Arrow is pointing to the hemorrhage in the dermis.

Histological evaluation of both skin biopsies revealed multifocal dermal expansion with homogenous amphophilic material (injection product) admixed with foci of extravasated red blood cells and clear space, consistent with hemorrhage and edema, respectively. Scattered dermal vessels were disrupted by the amphophilic material and a small amount of cellular debris and degenerate neutrophils; a single vessel contained fibrin with embedded monocytes that was adhered to the intima, consistent with a thrombus. There was no visible clinical necrosis at any meperidine injection site area after the biopsy (Figure 3).

## Discussion

In this study, meperidine administration did not alter histamine, tryptase, or IgE concentrations at the timepoints analyzed.

In humans, IV meperidine causes an increase in histamine plasma concentrations as early as 1 min following injection and lasting 6.5 min post injection. In these patients, signs of shock (hypotension, tachycardia) with erythema and a catecholamine surge were documented (24). In anesthetized dogs administered meperidine at 5 mg/kg IV, histamine concentrations increased 4–5 fold 1 min following injection and decreased to two to three times the baseline values after 5 min (30). Following the administration of 10 mg/kg IV meperidine, dogs developed immediate cutaneous and systemic reactions. As reactions were blunted by exhaustion of histamine stores when dogs were pre-treated with a mast cell degranulator, the adverse effects caused by meperidine were attributed to histamine release (23).

There are three possible reasons to explain why no increase in plasma histamine was observed in the current study. First, it is possible that the increase in histamine peaked and lasted <5 min. The authors chose a first sampling time point of 5 min in order to capture the window of elevation previously observed in dogs (30) and humans after IV injection (24). Typically, return to baseline of histamine plasma concentrations occurs 15–60 min post injection (31, 32). However, as the treatments administered in the present study were IM and SC, the authors expected a delayed rise in histamine compared to IV. Second, there is a potential that we sampled too early or that the effect on histamine occurred between timepoints. Lastly, it is possible that no histamine release occurred in these horses.

No significant change in plasma tryptase from baseline was noted at any timepoint. This differs from reports in humans, where various opioids including meperidine, morphine, codeine, and heroin produced elevations of tryptase (9, 33, 34). During pseudoallergy reactions to medications, including opioids, elevated tryptase levels are detected as early as 10 min following drug administration and up to 12 h post exposure (9). In humans administered IV heroin, tryptase plasma concentrations were elevated an hour following drug administration (34). Based on the human literature, circulating tryptase levels are ideally measured 1–2 h following the initiation of a systemic reaction (35) and can be elevated for as long as 48 h (36). The sensitivity and specificity of the tryptase test are reportedly poor (37), so the absence of changes in tryptase may be a result of the test itself. The lack of effect on plasma tryptase observed here also could have been attributed to the same reasons outlined above for histamine; sampling too early, too late, or there was truly no effect on plasma tryptase.

Anaphylaxis can occur following prior exposure and development of IgE to a substance or one with a similar chemical structure (31). Anaphylactic reactions to opioids are rare in people, but they are associated with severe complications and higher mortality (38, 39). True anaphylactic reactions during the perioperative period in humans are estimated to occur in 5–30 in 100,000 cases (35) and have documented elevations of histamine and prostaglandins confirmed within 30–60 min of the adverse reaction (31, 40). Serum IgE concentrations in the current study were consistent with previously reported values in horses (41). The lack of effect on serum IgE concentrations in the current study confirmed the authors' hypothesis. It is highly unlikely, given the rarity with which true anaphylactic reactions are reported after opiate administration, that we would have been able to document increases in the production of IgE in such a small group of horses. Additionally, one significant limitation to this study is the lack of meperidine-specific IgE measurement. The authors are not aware of any commercially available assays for measurement of opiate-specific immunoglobulins in horses, and recognizing this limitation, elected to evaluate total serum IgE. It is presumed that this group of research horses has never been previously exposed to meperidine, and small increases in the production of opiate-specific IgE may not have been detectable relative to total serum IgE concentrations. Regardless of the aforementioned limitations, based on the lack of systemic clinical signs consistent with anaphylaxis, and the lack of increase in histamine, tryptase, and serum IgE concentrations, no evidence of an IgE-mediated event was identified in these horses.

To the authors' knowledge, the current study is the first to report a localized reaction to SC injection of meperidine in healthy horses. Historically, diffuse sweating has been noted with the administration of opioids in horses, rats, and humans. This reaction is considered to be centrally mediated due to alteration of the temperature set point (42–44). Generalized sweating induced by meperidine has been previously reported after IV administration (21). However, the sweating observed in these horses was localized and therefore unlikely to be caused by a centrally mediated process. Cutaneous injections of meperidine, like other opioids, have been shown to induce wheal reactions in humans and cats due to histamine release (2, 45, 46). In the current study, however, a lack of systemic increase in histamine concentrations suggests that a different mechanism may have occurred, albeit tissue histamine within the biopsies was not quantified. For example, other inflammatory mediators known to cause swelling after intradermal injection in horses, such as bradykinin (47), may have been involved.

Amphophilic material, likely the injected product, present in the cutaneous biopsies might have caused a local inflammatory reaction that induced the wheal formation with disruption of the dermal vessels, mild vascular necrosis, and thrombosis resulting in leaky vessels and the localized sweating. It is impossible to establish whether this reaction was induced by the meperidine or the buffer, acetic acid sodium acetate, present in the product used. Meperidine injections given IV have been shown to induce phlebitis and thrombosis in humans (48), whereas subcutaneous administrations induced skin necrosis at the injection sites in rats (49). The vascular disruption in the current study was considered mild, as no macroscopic necrosis was observed in the horses. Due to the fact that this reaction occurred to varying extents in each horse receiving SC meperidine and the long term effects are unclear, the subcutaneous administration of meperidine to horses clinically may be discouraged. There was no evidence of regional sweating or wheal-like lesions appreciated at the IM injection sites; however, these were not biopsied. Therefore, the occurrence of an injection site reaction at the cellular level cannot be ruled out through this route of administration. However, in the authors' opinion, IM injection may be preferrable due to the lack of sustained wheal-like lesions as compared to SC administration.

There were several limitations to this study. As anaphylactic or pseudoallergy reactions can be variable and rare events, the occurrence of a histamine, tryptase, or IgE-mediated event may not have been detected due to the small sample size. For example, the achieved power for treatment in a *post-hoc* analysis (*F* test, alpha 0.05, sample size 6, groups 3, and effect size f 0.523, G^*^Power 3.1.9.4) for the histamine was only 14.6%. Additionally, the timepoints at which these blood concentrations were obtained may not have captured an acute rise in concentrations of these substances. A sham treatment was not included to analyze the buffer, acetic acid sodium acetate, present in the meperidine solution tested. However, the formulation used is approved for SC, IM, or slow IV routes of administration in humans and to the authors' knowledge, acetic acid sodium acetate has not been associated with cutaneous reactions. Lastly, histamine was not quantified in the biopsy samples, and it is possible that a local histamine reaction could have occurred at the site of the subcutaneous injection. This possibility should be taken into consideration for future studies related to subcutaneous administration of meperidine in horses.

## Conclusions

No changes in plasma histamine, tryptase, or serum IgE concentrations were noted in any of the horses in this study. Therefore, neither an anaphylactoid or anaphylactic reaction was confirmed. Administration of SC meperidine caused localized vasculitis, thrombosis, regional edema, and hemorrhage.

## Data Availability Statement

The raw data supporting the conclusions of this article will be made available by the authors, without undue reservation.

## Ethics Statement

The animal study was reviewed and approved by University of Georgia's Institutional Care and Use Committee.

## Author Contributions

RR conceived the study. HK, RR, JQ, and MB designed the study. HT, LB, AH, CR, and RR collected data. BM and FB collected samples. LB, HK, BM, FB, and RR processed samples. DS analyzed data. HT wrote the manuscript. Results were interpreted and the manuscript was critically revised by HT, DS, LB, AH, HK, CR, BM, FB, JQ, MB, and RR. All authors read and approved the final manuscript.

## Conflict of Interest

The authors declare that the research was conducted in the absence of any commercial or financial relationships that could be construed as a potential conflict of interest.

## Abbreviations

IgE: immunoglobulin E
IV: intravenous
IM: intramuscular
SC: subcutaneous
ACN: acetonitrile
LC-MS/MS: liquid chromatography tandem mass spectrometry
ELISA: enzyme-linked immunoabsorbent assays.
